# A155 OUTCOMES FOLLOWING ENDOSCOPIC RESECTION OF DUODENAL NEUROENDOCRINE TUMOURS FROM A TERTIARY-CARE ACADEMIC CENTRE

**DOI:** 10.1093/jcag/gwac036.155

**Published:** 2023-03-07

**Authors:** S Gupta, G Brar, K Zheng, S Hakim, C W Teshima, G R May, C H Law, J Hallet, J D Mosko

**Affiliations:** 1 Medicine, University of Toronto; 2 Laboratory Medicine and Pathobiology, St. Joseph’s Health Centre; 3 The Center for Advanced Therapeutic Endoscopy and Endoscopic Oncology, St. Michael’s Hospital; 4 Surgery, Sunnybrook Health Sciences Centre, Toronto, Canada

## Abstract

**Background:**

Duodenal neuroendocrine tumours (D-NET) are rare cancers derived from neuroendocrine cells of the duodenum. A steady increase in the incidence of these tumours has been observed. Current treatment and surveillance strategies are guided by various tumour characteristics including size, grade, and depth of invasion. There exists conflicting evidence, however, on the rates of recurrence after positive resection margins following endoscopic resection. Thus, it remains uncertain whether complete endoscopic resection (R0) of these indolent tumours is clinically significant and whether follow-up endoscopic or surgical intervention is justified.

**Purpose:**

Our aim is to characterize endoscopic management and clinical outcomes in patients undergoing endoscopic resection of D-NETs.

**Method:**

We conducted a retrospective, single-centre cohort study at The Centre for Advanced Therapeutic Endoscopy and Endoscopic Oncology at St. Michael’s Hospital, Toronto, Ontario. Consecutive patients over the age of 18 who underwent endoscopic resection of histologically proven D-NETs between 2011 and 2020 were included. Data on patient, endoscopic, and tumour characteristics were collected through electronic chart review. Descriptive statistics were conducted for data analysis.

**Result(s):**

A total of 155 foregut neuroendocrine tumours (NET) were endoscopically resected amongst 96 patients during the study period. 47 of these were histologically identified as D-NETs. Mean tumour size was 9.88 ± 6.86 mm. Conventional endoscopic mucosal resection (EMR) was performed most frequently (55%, n=26/47), followed by cap-assisted EMR (30%, n=14/47). Hybrid endoscopic submucosal dissection (ESD)/EMR was performed in one case. A total of two intra-procedural perforations occurred, both of which were successfully closed endoscopically. One patient with a peri-ampullary D-NET experienced significant intra-procedural bleeding requiring Hemospray® and subsequent endotracheal intubation resulting in a brief hospitalization. 57% of all resected D-NETs were followed at surveillance endoscopy 1 (SE1) at a median interval of 199 days (range, 84 to 830). Positive resection margins (R1) were found in 26 cases (55%), of which 16 were assessed at SE1 while nine were lost to follow-up. One patient with R1 margins was electively treated with APC at SE1. Tumour recurrence at SE1 occurred in only two patients.

**Image:**

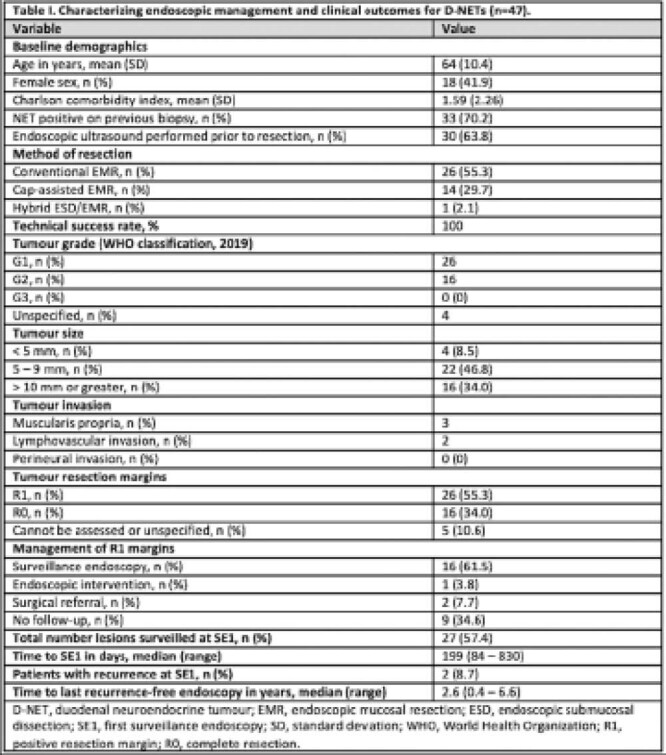

**Conclusion(s):**

D-NET recurrence is found in less than 5% of patients at surveillance endoscopy following endoscopic resection in spite of a high R1 resection rate. Given this indolent nature of these tumours, our study suggests that patients with positive resection margins can be followed conservatively with surveillance endoscopy. Further investigation is warranted to determine the optimal duration and surveillance strategy for these patients.

**Please acknowledge all funding agencies by checking the applicable boxes below:**

CAG

**Disclosure of Interest:**

None Declared

